# Icariin reduces human colon carcinoma cell growth and metastasis by enhancing p53 activities

**DOI:** 10.1590/1414-431X20187151

**Published:** 2018-08-06

**Authors:** Meili Tian, Shuang Yang, Xinpeng Yan

**Affiliations:** 1 Department of Health Care, Shengli Oilfield Central Hospital, Dongying, Shandong, China; 2 Department of Health Management, Shengli Oilfield Central Hospital, Dongying, Shandong, China; 3 Department of Traditional Chinese Medicine, Shengli Oilfield Central Hospital, Dongying, Shandong, China

**Keywords:** Colon carcinoma, Icariin, Mechanism, p53, DNA damage, Caspases

## Abstract

Icariin has been reported to possess high anticancer activity. Colon carcinoma is one of the leading causes of cancer-related mortality worldwide. Here, the anticancer activity of icariin against HCT116 colon carcinoma cells and the possible underlying mechanism were studied. The trypan blue staining assay, wound healing assay, clonogenic assay, CCK-8 assay, and Annexin V-FITC/PI double staining method were carried out to determine the changes of HCT116 cell growth and migration. mRNA and protein expressions were determined by quantitative real-time PCR and western blot, respectively. Moreover, small interfering RNA (siRNA) plasmid was used to examine the role of p53 in icariin-induced apoptosis in HCT116 cells. Icariin significantly suppressed colon carcinoma HCT116 cells by decreasing migration and viability, and simultaneously promoting apoptosis. Icariin exerted the anti-tumor effect in a dose-dependent manner by up-regulating p53. During treatment of icariin, p-p53, p21, and Bax levels increased, and Bcl-2 level decreased. Short time treatment with icariin induced DNA damage in HCT116 cells. Furthermore, the cytotoxicity of icariin was decreased after p53 knockdown or by using caspase inhibitors. p53 was involved in activities of caspase-9 and caspase-3. Icariin repressed colon carcinoma cell line HCT116 by enhancing p53 expression and activating p53 functions possibly through Bcl-2/Bax imbalance and caspase-9 and -3 regulation. Icariin treatment also induced DNA damage in HCT116 cells.

## Introduction

Colon carcinoma is a common type of malignant tumor of the alimentary system, with high morbidity and mortality. In recent years, the incidence of colon carcinoma has increased worldwide. Due to increasing resistance to conventional treatment, new therapies should be urgently explored (1).

Icariin, a flavonol glycoside and a major constituent of the extract from leaf and stem of *Herba epimedii* (Berberidaceae) plant, has been found to have antineoplastic activities against a variety of human malignancies (2,3). As a tumor inhibitor, icariin has been shown to inhibit cell growth by arresting cells in G1 phase and decreasing mitochondrial transmembrane potential in prostate carcinoma cells (4). Icariin also exerted its negative effects on human gastric cancer cell invasion and migration by vasodilator-stimulated phosphoprotein via Rac1 pathway (5), and regulated the proliferation and apoptosis of human ovarian cancer cells through microRNA-21 by targeting some tumor suppressor genes (6). Icariin showed high potential of anti-tumor effect on many cancer cells and the anticancer mechanisms have been widely researched. However, the biological role of icariin in colon carcinoma and its underlying molecular mechanism remain undefined.

Some studies reported that the transcriptional factor p53 played an indispensable role in active function of icariin (7,8). p53 is one of the most important tumor suppressors in cells, which can protect normal cell growth and initiate malignant cell death. In unstressed cells, the level and activity of p53 is strictly controlled especially by the ubiquitin E3 ligase mdm2 (9). Blocking the mdm2-p53 interaction and reactivating p53 function is a promising therapeutic strategy for the treatment of cancers (10). p53 can be activated when cells suffer toxic stresses, inducing cell growth arrest, cell senescence, and apoptosis (11,12). Thus the functions of p53 in icariin-treated cells were analyzed.

In this study, the anti-tumor effect of icariin in human colon carcinoma cells was assessed. The interaction between icariin and p53 was also investigated in order to reveal the underlying action mechanisms of icariin and the role of p53 in the anti-tumor effect of icariin.

## Material and Methods

### Cell culture and transient transfection

Human colon carcinoma HCT116 cells and normal colon epithelial FHC cells were obtained from the American Type Culture Collection (USA). Cells were cultured in Dulbecco's modified Eagle medium (Hyclone, USA) and supplemented with 10% fetal calf serum (Hyclone), in a humidified incubator containing 95% air and 5% CO_2_ at 37°C. The specific siRNA against p53 was purchased from Santa Cruz Biotech (USA). Transfections were carried out using Lipofectamine 2000 reagent (Invitrogen, USA) according to instructions. After 48 h of transfection, cells were harvested for analyses.

### Cell viability assay

HCT116 cells were plated in 24-well plates at a density of 1×10^5^ cells per well and then treated with various doses of icariin (Shanghai U-sea Biotech Co., Ltd., China). Wells added with DMSO were used as negative controls. Cells were trypsinized and stained with trypan blue dye, and viable cells were counted using a cell counting chamber every day for a total of 5 days. Finally, cell growth curves were plotted according to the viable cell numbers of each group. The viabilities of the HCT116 and FHC cells after icariin treatment were assessed by Cell Counting Kit-8 (CCK-8, Dojindo Molecular Technologies, USA), and the difference between them was analyzed. HCT116 cells were seeded in a 96-well plate at a density of 5×10^3^ cells per well. After icariin treatment, 20 μL CCK-8 solution was added to the culture medium and the cultures were further incubated for 1 h at 37°C in humidified 95% air and 5% CO_2_. After incubation, the absorbance was measured at 450 nm using a Microplate Reader (Bio-Rad, USA).

### Wound healing assay

A wound healing assay was conducted to evaluate the migratory capacity of HCT116 cells in each group. Equal numbers of cells were cultured to 95% confluence in 6-well plates. The wound gaps were created by scratching cell sheets with a sterile 200 μL-pipette tip. Wells were washed twice with PBS to remove the floating cells and added with fresh mediums containing DMSO or icariin with final concentrations of 50 and 100 nM. Changes of the scratched areas were observed by an inverted microscope (Leica, Germany) every 24 h. Wound widths were measured to calculate the relative widths.

### Clonogenic assay

HCT116 cells were plated in 60-mm dishes (1000 cells per well) and cultured in a medium containing DMSO or icariin. After 2 weeks, cell clones were fixed with a 4% paraformaldehyde solution (Beyotime Biotechnology, China) and stained with 0.1% crystal violet (Beyotime Biotechnology). Visible colonies on plates with different treatments were captured by ChemiDoc XRS+ imaging system (Bio-Rad). The surviving fraction was calculated as a ratio of the number of colonies to the number of plated cells (plating efficiency), divided by the same ratio calculated for the non-treated group.

### Apoptosis assay

Annexin V-FITC/PI apoptosis detection kit (Beijing Biosea Biotechnology, China) was used for apoptosis analysis. Briefly, cells were seeded on 6-well plates (1×10^5^ cells/well). Treated cells were washed twice with PBS and resuspended in a binding buffer (KeyGEN Biology, China). The adherent and floating cells were collected and treated following the manufacturer's protocol, and samples were immediately analyzed on a flow cytometer (Beckman Coulter, USA) to differentiate apoptotic cells (Annexin-V positive and PI-negative) from necrotic cells (Annexin-V and PI-positive).

### Real-time PCR

HCT116 cells were treated with various concentrations of icariin for 24 h. Total RNA was then extracted using the RNAiso Plus Reagent (TaKaRa, China) according to the manufacturer's instructions. Total RNA with 500 ng was reversely transcribed to cDNA using PrimeScriptTM RT Master Mix (Perfect Real Time (TaKaRa). Real-time PCR was performed on the CFX 96 Real-time PCR detection system (Bio-Rad) using SYBR® Premix Ex TaqTM II (Tli RnaseH Plus) and specific primers (TaKaRa). The mRNA level of each gene was normalized to β-actin with 2^-ΔΔCt^ method using Bio-Rad CFX Manager V1.1.308.1111 software.

### Western blot

The proteins were extracted using RIPA lysis buffer (Beyotime Biotechnology) supplemented with protease inhibitors (Roche, Switzerland), and quantified using the BCA™ Protein Assay Kit (Pierce, USA). The western blot system was established using the Bio-Rad Bis-Tris Gel system (Bio-Rad) according to the manufacturer's instructions. The cell lysates were boiled in a 5 X SDS-PAGE loading buffer (Beyotime, China) for 10 min, resolved by 8% SDS-PAGE, and transferred to a polyvinylidene difluoride (PVDF) membrane. After a blocking incubation with 5% milk-TBST, the membranes were incubated with primary antibodies at a dilution of 1:1000 for the specific detection of caspase-3 (ab4051), cleaved caspase-3 (ab32042), caspase-9 (ab32539), cleaved caspase-9 (ab32539), PARP (ab32138), cleaved PARP (ab32138), p53 (ab131442), mdm2 (ab38618), p-p53(ab1431), p21 (ab109520), Bcl-2 (ab32124), Bax (ab32503), γ-H2AX (ab11174), and β-actin (ab8227, Abcam, USA) at 4°C overnight. The membranes were then incubated a third time with an appropriately correlated secondary antibody (1:2000 dilution, Abcam) that was conjugated to horseradish peroxidase. After rinsing, the PVDF membrane-carried blots and antibodies were transferred into the Bio-Rad ChemiDoc™ XRS system, with an addition of 200 μL Immobilon western chemiluminescent HRP substrate (Millipore, USA) to cover the membrane surface. The signals were captured and the intensity of the bands was quantified using Image Lab™ Software (Bio-Rad).

### Statistical analysis

All experiments were repeated three times. Results are reported as means ±SD. Statistical analyses were performed using Graphpad statistical software (GraphPad Software Inc., USA) and P values were calculated using a one-way analysis of variance (ANOVA). P<0.05 was considered to be significant.

## Results

### Icariin inhibited the growth and migration of colon carcinoma cells

**Figure 1.**
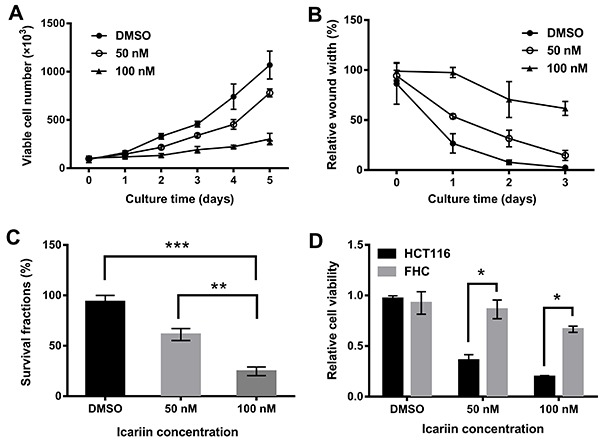
Growth and migration of HCT116 cells were suppressed by icariin and the inhibitory effect was both time- and dose-dependent. *A*, Viability and (*B*) migration of HCT116 cells were assessed by the trypan blue staining method and wound healing assay, after treatment with different dosages of icariin for different times. *C*, Colony formation was assessed for viability analysis. *D*, Viability of HCT116 cells and FHC (normal) cells after icariin treatment were compared according to CCK-8 assay. Assays were performed in triplicate. Data are reported as means±SD. *P<0.05, **P<0.01, ***P<0.001 (ANOVA).

The effect of icariin on HCT116 cell viability was specifically analyzed. The results showed that viability was inhibited, with an enhanced effect after an increase in icariin concentration and extension in treatment time (Figure 1A). Next, the effect of icariin on cell migration was detected by a wound healing assay. As shown in Figure 1B, icariin at 50 and 100 nM effectively suppressed cell migration in a time-dependent manner, compared with DMSO control. Meanwhile, a clonogenic assay was carried out to analyze the effect of icariin on HCT116 cell growth. Compared with the number of colonies in the DMSO control group, those in icariin-treated groups decreased significantly, suggesting that icariin markedly suppressed HCT116 cell growth (Figure 1C). Moreover, according to the CCK-8 assay, icariin significantly reduced the viability of HCT116 cells compared to normal colon FHC epithelial cells (P<0.05; Figure 1D), indicating that HCT116 cells were more sensitive to icariin.

### Icariin promoted apoptosis of colon carcinoma cells

**Figure 2.**
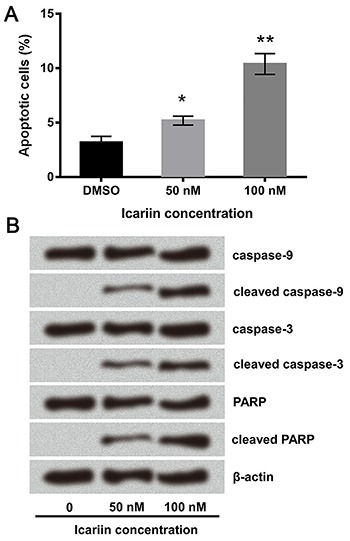
Icariin induced apoptosis of colon carcinoma cells. *A*, Apoptotic analysis of HCT116 cells. HCT116 cells were treated with different doses of icariin and incubated with AV-FITC and PI. Percentage of apoptotic cells (AV-FITC +/PI−) was obtained using flow cytometry. *B*, Western blot analysis of caspase-9 and -3 and PARP proteins in icariin-treated cells. β-actin was used as internal control. Assays were performed in triplicate. Data are reported as means±SD. *P<0.05, **P<0.01 (ANOVA)

The anti-tumor efficacy of icariin was further evidenced by the detection of cell apoptosis. The flow cytometry analysis given in Figure 2A displayed that apoptotic cell rates significantly increased after icariin treatments of 50 nM (P<0.05) and 100 nM (P<0.01), which is consistent with the western blot assay data (Figure 2B). Some apoptosis-promoting proteins were activated by icariin, including cleaved caspase-9 and -3 and cleaved PARP.

### Icariin enhanced the anti-tumor effect of cisplatin

**Figure 3.**
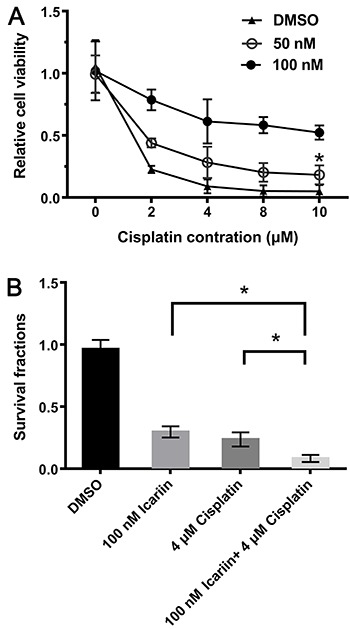
Icariin enhanced the anti-tumor effect of cisplatin on colon carcinoma cells. The effect of icariin and cisplatin in combination on cell growth was detected by *A*, CCK-8 assay and *B,* colony formation assay. Assays were performed in triplicate. Data are reported as means±SD. *P<0.05 (ANOVA).

Since cisplatin is one of the main clinical therapies for cancer, we evaluated if it could be used in combination with icariin to treat colon carcinoma, or if icariin could enhance the sensitivity of colon carcinoma cells to cisplatin. As shown in Figure 3A, the combination of icariin and cisplatin inhibited tumor cell viability more effectively than their respective treatments separately. In the clonogenic survival assay, the combination significantly enhanced HCT116 cell mortality (P<0.05; Figure 3B).

### p53 was up-regulated in icariin-treated cells

**Figure 4.**
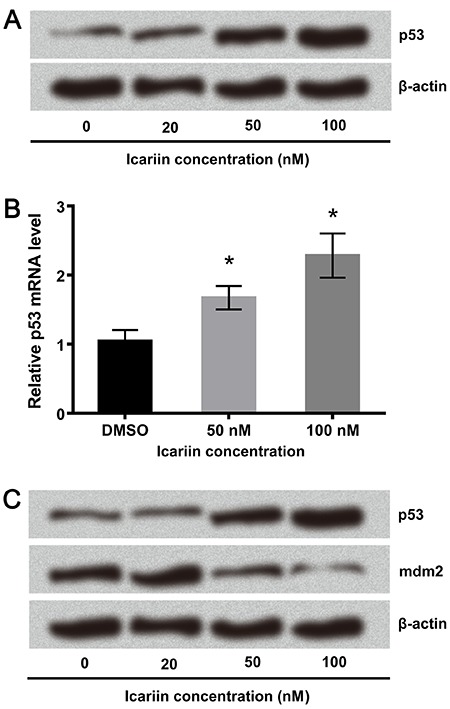
Expression of p53 was enhanced by icariin. *A*, The mRNA level of p53 was analyzed by real-time PCR. *B* and *C*, Protein levels of p53, p-p53, and mdm2 were detected by western blot analysis. β-actin was used as internal control. Assays were performed in triplicate. Data are reported as means±SD. *P<0.05 (ANOVA).

The anti-colon carcinoma effect of icariin was verified, but the underlying mechanism was unclear. HCT116 cells express p53, an important tumor suppressor, the overexpression of which can lead to cell cycle arrest and apoptosis. Therefore, we speculated that p53 might be involved in the carcinostatic role of icariin. According to the results, p53 was indeed up-regulated, and mRNA and protein levels of p53 were enhanced along with the increase in icariin concentration (Figure 4A and B). Phosphorylation of the NH_2_-terminal residues of p53 could mediate its stabilization and nuclear accumulation after treatment with anticancer drugs (13). Thus, p-p53 level gradually increased when treated with equally increasing concentrations of icariin. In normal, non-transformed cells, p53 levels are kept low through activities of negative regulators, such as mdm2 (14). Some studies reported that under stress and with the inhibition of mdm2/p53 interaction, p53 protein levels rapidly increased (15,16). In this study, we found that mdm2 was down-regulated after icariin treatment, which was accordingly disproportionate to p53 level. Therefore, p53 might be targeted by mdm2 in icariin-treated cells.

### Effect of icariin on cell growth-related proteins in colon carcinoma cells

**Figure 5.**
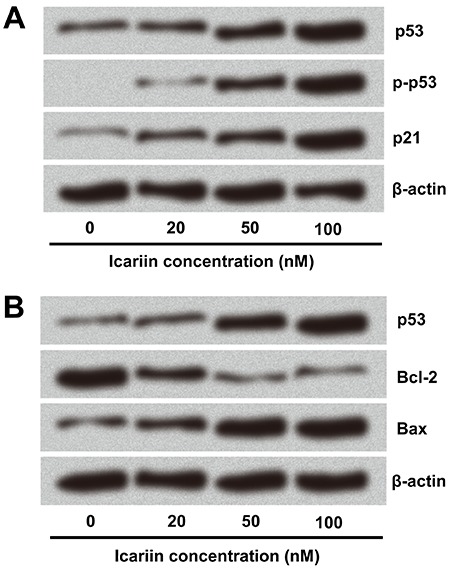
Effect of icariin on the cell growth-related proteins in colon carcinoma cells. The levels of p21 (*A*), Bcl-2, and Bax (*B*) expressions were detected by western blot. β-actin was used as internal control.

p21 was the key p53 target protein, which was enhanced after icariin treatment (Figure 5). Next, levels of important apoptosis-related proteins Bcl-2 and Bax were also evaluated. The anti-apoptosis protein Bcl-2 was down-regulated by icariin in a dose-dependent manner, while the pro-apoptosis protein Bax level was increased (Figure 5). These data suggested that icariin not only enhanced p53 expression, but possibly also regulated other important genes interfering with cell growth.

### Icariin induced DNA damage in colon carcinoma cells

**Figure 6.**
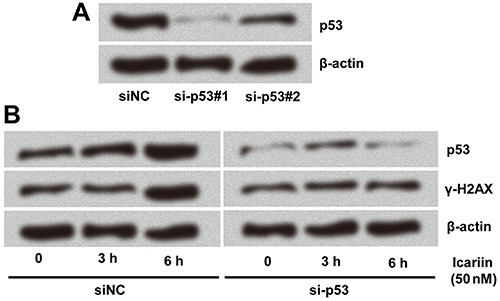
Icariin induced DNA damage in HCT116 cells. *A*, The protein levels of p53 were analyzed after transfection with two different sequences of p53 targeted siRNAs (si-p53 #1 and si-p53 #2). *B*, Effect of p53 knockdown on the induction of DNA damage by icariin. Data were obtained through western blot. β-actin was used as internal control.

p53 is a key regulator not only of the cell cycle and apoptosis, but also of DNA repair. Due to the abnormal expression of p53, we hypothesized that icariin might induce DNA damage. We evaluated the effect of icariin on the level of γ-H2AX (phosphorylated histone H2AX on serine 139, a sensitive DNA damage marker specifically induced by DNA double-strand breaks). Firstly, we constructed the specific siRNA against p53, to silence p53 in HCT116 cells. p53 was successfully knocked down after transfection with siRNA #1 and siRNA #2 (Figure 6A). The si-p53 #1 was applied in the next experiment. As shown in Figure 6B, γ-H2AX was up-regulated by icariin after treatment for 6 h, confirming the DNA damage property of icariin. Subsequently, the effect of icariin on DNA damage after p53 knockdown was investigated, and results showed that γ-H2AX expression was not enhanced. This indicated that p53 knockdown inhibited icariin-induced DNA damage and DNA damage was an outcome of p53-induced apoptosis (Figure 6B).

### Icariin inhibited viability and promoted apoptosis of colon carcinoma cells through p53-activated caspase-9 and -3

**Figure 7.**
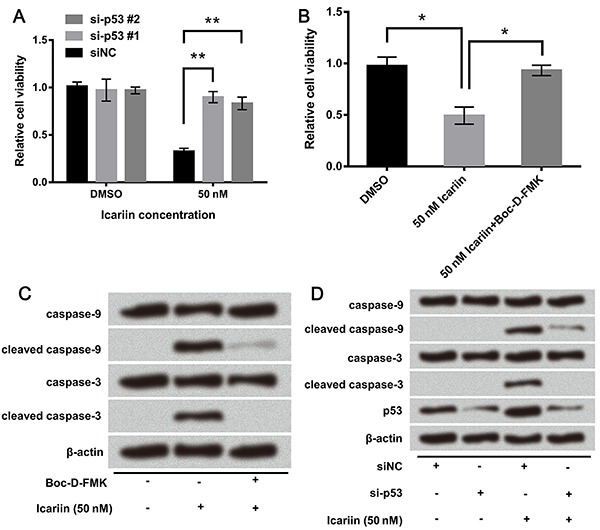
p53 was involved in the anti-tumor effect of icariin by activating caspase-9 and -3. *A*, Effect of p53 knockdown on cell viability. *B*, Effect of caspases inhibitor (Boc-D-FMK) on cell viability. Cell viability was determined by CCK-8 assay. *C*, The effect of caspase inhibitors and *D*, p53 knockdown on protein levels of caspase-9 and -3 were analyzed by western blot. β-actin was used as internal control. Data are reported as means±SD. *P<0.05, **P<0.01 (ANOVA)

In this study, the role of p53 in icariin-treated colon carcinoma cells was assessed further. Firstly, cell viability was detected when p53-targeted siRNA was transfected into icariin-treated cells. As shown in Figure 7A, the si-p53 #1 and si-p53 #2 abolished the viability-inhibiting effect of icariin. Similar results were found in the treatment group with the addition of Boc-D-FMK (a caspase inhibitor) (Figure 7B), which abolished icariin-induced decrease in cell viability. The western blot assay displayed that high levels of cleaved caspase-9 and -3 induced by icariin were decreased by the addition of Boc-D-FMK, indicating that icariin promoted apoptosis of HCT116 cells by activating caspase-9 and -3 (Figure 7C). As shown in Figure 7D, caspase-9 and -3 were not activated by icariin after p53 knockdown. The data identified p53-dependent caspase-mediated mechanisms leading to apoptosis during treatment period with icariin. In summary, caspase-9 and -3 played an important role in the anti-tumor effect of icariin and were regulated by p53.

## Discussion

Colorectal cancer is among the most common malignancies, and has been rising as one of the main diseases threating public health and quality of life. Its mortality rate is only inferior to gastric carcinoma, esophageal carcinoma, and primary colon cancer, ranking forth in malignant tumors of the digestive system. Although colorectal cancer diagnosis and treatment with chemoradiation (alone or in combination with surgery) has greatly improved, it remains a problem that would benefit from more effective therapeutic strategies.

In the present study, we evaluated the anticancer activity of icariin against colon cancer cell line HCT116. The results indicated that growth, migration, colony formation, and viability of HCT116 cells were inhibited by icariin and the suppressing effect was improved with the increase of icariin concentration. Moreover, icariin was found to promote cell apoptosis in a dose-dependent manner, and some apoptosis-promoting proteins were enhanced after icariin treatment. The obtained results were consistent with other reports. Research showed that icariin induced cytotoxicity with an IC_50_ of 40 μM in esophageal cancer cells, induced G2/M cell cycle arrest, and inhibited esophageal cancer cell migration, invasion, and metastasis (17). It also exerted negative effects on human ovarian and gastric cancer, among others (3,6).

Our results showed that the combination of icariin with cisplatin provided more effective anti-tumor activity compared to treatment with icariin or cisplatin alone. Previous studies demonstrated that icariin enhanced the *in vitro* antitumor activity of arsenic trioxide against acute promyelocytic leukemia (18), as well as the cytotoxicity of doxorubicin in human multidrug-resistant osteosarcoma cells (19). These findings suggest that a combination of icariin with other anticarcinogens might offer a therapeutic benefit to patients with cancer.

Icariin was considered a potential drug for colorectal cancer therapy based on its strong anti-tumor effect, which led to the assessment of the possible underlying mechanisms. Our findings showed that p53 played an important role in the action mechanism of icariin. p53 expression was enhanced both in mRNA and protein levels when HCT116 cells were treated with icariin. Furthermore, p53 overexpression might be caused by abnormal expression of icariin-induced mdm2. The mdm2 can directly bind to p53 and continuously mediate p53 ubiquitination and proteasomal degradation. Besides, p53 level and activity is strictly controlled by mdm2 (20,21). The present study showed that nutlin-bound mdm2 disturbed the p53-mdm2 interaction, and then showed anti-tumor activity by activating p53 (22).

The p53 gene is important for apoptosis regulation (23). Hence, apoptosis is suppressed if the p53 gene is mutated or its function is damaged. Wild type p53 is known to be a regulator of Bax, because a p53-binding site has been found in the Bax gene promoter (24). Therefore, p53 is responsible for regulating cell death through Bcl-2/Bax imbalances, which was consistent with our data; down-regulation of Bcl-2 and up-regulation of Bax were found in our study. Additionally, previous studies also reported the role of p53 in HCT116 cells, which showed that cell-cycle arrest in G1 and G2 were regulated by p53 (25), and p53-wild type participated in HCT116 colon carcinoma cell apoptosis, leading to caspase activation and cell death (26). These studies indicated that targeting p53 might be an effective strategy to fight colon carcinoma.

Cancer therapy drugs such as cisplatin, mitomycin C, etoposide, and a number of other compounds are known to act on cellular DNA (27). Thus, the effect of icariin on cellular DNA was also investigated. Results displayed DNA damage occurring at 6 h after icariin treatment (50 nM), but the damage was inhibited after p53 silencing. It might be possible that p53 knockdown inhibited the cytotoxicity of icariin and impaired the harmful effect on HCT116 cells, including DNA damage. However, the detailed underlying mechanism requires further study.

Based on our results, icariin exerted its anti-tumor effect by regulating caspase-9 and caspase-3, but the caspases could not be activated by icariin after p53 knockdown. Therefore, caspase-9 and caspase-3 might be directly regulated by p53, and p53 by icariin. Some studies reported that icariin inhibited colorectal cancer cell growth by mainly suppressing NF-κB activity (28,29). However, the pharmacological effects of anti-cancer drugs are particularly complex and their effects might occur in more than one way. Therefore, more studies are needed in future.

In summary, our study showed that icariin effectively inhibited migration and viability of the colon carcinoma cell line HCT116 and promoted apoptosis. Icariin had an anti-tumor effect in a p53-dependent manner and p53 might be regulated by mdm2. Icariin may represent a promising therapeutic strategy for the treatment of human colon carcinoma.
